# Cyprinid Herpesvirus 3

**DOI:** 10.3201/eid1612.100593

**Published:** 2010-12

**Authors:** Benjamin Michel, Guillaume Fournier, François Lieffrig, Bérénice Costes, Alain Vanderplasschen

**Affiliations:** Author affiliations: University of Liège, Liège, Belgium (B. Michel, G. Fournier, B. Costes, A. Vanderplasschen);; Centre d'Economie Rurale Groupe, Marloie, Belgium (F. Lieffrig)

**Keywords:** Cyprinid herpesvirus 3, CyHV-3, koi herpesvirus, KHV, Alloherpesviridae, common carp, koi carp, fish, viruses, synopsis

## Abstract

The recently designated cyprinid herpesvirus 3 (CyHV-3) is an emerging agent that causes fatal disease in common and koi carp. Since its emergence in the late 1990s, this highly contagious pathogen has caused severe financial losses in common and koi carp culture industries worldwide. In addition to its economic role, recent studies suggest that CyHV-3 may have a role in fundamental research. CyHV-3 has the largest genome among viruses in the order Herpesvirales and serves as a model for mutagenesis of large DNA viruses. Other studies suggest that the skin of teleost fish represents an efficient portal of entry for certain viruses. The effect of temperature on viral replication suggests that the body temperature of its poikilotherm host could regulate the outcome of the infection (replicative vs. nonreplicative). Recent advances with regard to CyHV-3 provide a role for this virus in fundamental and applied research.

## Abstract 2

TOC summary: This virus is useful for fundamental and applied research.

The common carp (*Cyprinus carpio carpio*) is a freshwater fish and one of the most economically valuable species in aquaculture; worldwide, 2.9 million metric tons are produced each year (1). Common carp are usually cultivated for human consumption. Koi (*C. carpio koi*) are an often-colorful subspecies of carp, usually grown for personal pleasure and competitive exhibitions. In the late 1990s, a highly contagious and virulent disease began to cause severe economic losses in these 2 carp industries worldwide (2) (Figure 1). The rapid spread was attributed to international fish trade and koi shows around the world (3). The causative agent of the disease was initially called koi herpesvirus because of its morphologic resemblance to viruses of the order Herpesvirales (3). The virus was subsequently called carp interstitial nephritis and gill necrosis virus because of the associated lesions (4). Recently, on the basis of homology of its genome with previously described cyprinid herpesviruses (5), the virus was assigned to family *Alloherpesviridae,* genus *Cyprinivirus*, species *Cyprinid herpesvirus 3* and renamed cyprinid herpesvirus 3 (CyHV-3). Because of the economic losses caused by this virus, CyHV-3 rapidly became a subject for applied research. However, recent studies have demonstrated that CyHV-3 is also useful for fundamental research. We therefore summarized recent advances in CyHV-3 applied and fundamental research.

**Figure 1:**
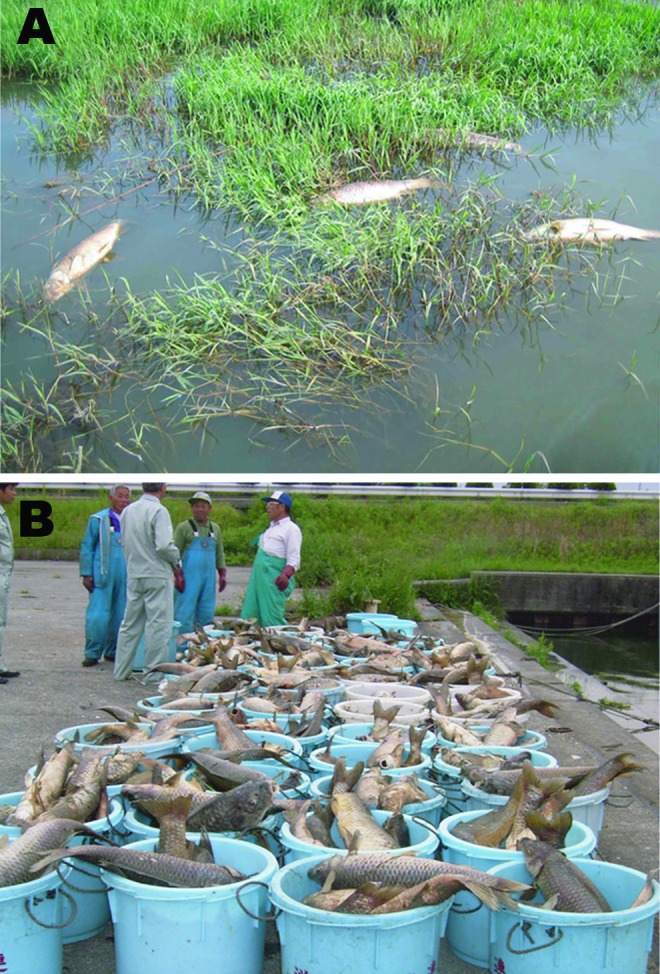
Mass deaths of common carp caused by cyprinid herpesvirus 3 infection in Lake Biwa, Japan, 2004. A) Dead wild common carp; deaths occurred throughout the lake. B) Dead carp (>100,000) collected from the lake in 2004. An estimated 2–3× more carp died but were not collected from the lake. Reproduced with permission from Matsui et al. (2).

## Characterization of CyHV-3

### Classification

**Figure 2:**
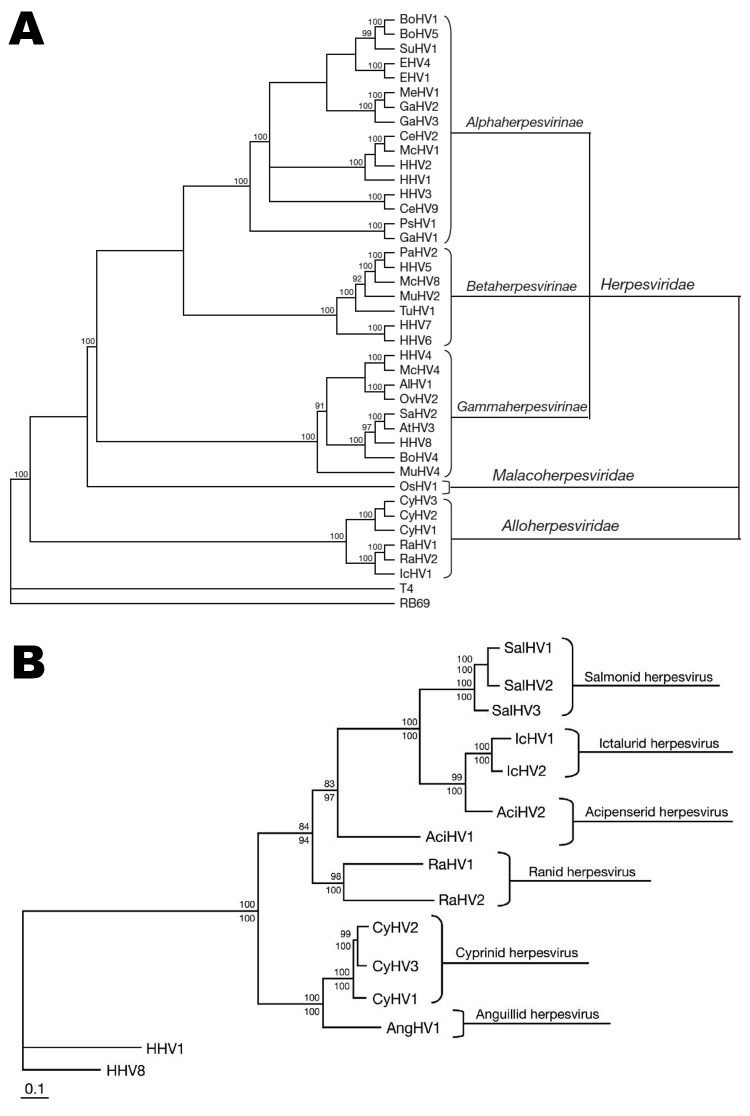
A) Cladogram depicting relationships among viruses in the order *Herpesvirales*, based on the conserved regions of the terminase gene. The Bayesian maximum-likelihood tree was rooted by using bacteriophages T4 and RB69. Numbers at each node represent the posterior probabilities (values >90 shown) of the Bayesian analysis. B) Phylogenetic tree depicting the evolution of fish and amphibian herpesviruses, based on sequences of the DNA polymerase and terminase genes. The maximum-likelihood tree was rooted with 2 mammalian herpesviruses (human herpesviruses 1 and 8). Maximum-likelihood values >80 and Bayesian values >90 are indicated above and below each node, respectively. Scale bar indicates branch lengths, which are based on the number of inferred substitutions. AlHV-1, alcelaphine herpesvirus 1; AtHV-3, ateline herpesvirus 3; BoHV-1, -4, -5, bovine herpesviruses 1, 4, 5; CeHV-2, -9, cercopithecine herpesviruses 2, 9; CyHV-1, -2, cyprinid herpesviruses 1, 2; EHV-1, -4, equid herpesvirus 1, 4; GaHV-1, -2, -3, gallid herpesvirus 1, 2, 3; HHV-1, -2, -3, -4, -5, -6, -7, -8, human herpesvirus 1, 2, 3, 4, 5, 6, 7, 8; IcHV-1, ictalurid herpesvirus 1; McHV-1, -4, -8, macacine herpesvirus 1, 4, 8; MeHV-1, meleagrid herpesvirus 1; MuHV-2, -4, murid herpesvirus 2, 4; OsHV-1, ostreid herpesvirus 1; OvHV-2, ovine herpesvirus 2; PaHV-1, panine herpesvirus 1; PsHV-1, psittacid herpesvirus 1; RaHV-1, -2, ranid herpesvirus 1, 2; SaHV-2, saimiriine herpesvirus 2; SuHV-1, suid herpesvirus 1; and TuHV-1, tupaiid herpesvirus 1. Adapted with permission from Waltzek et al. (6).

CyHV-3 is a member of the order Herpesvirales and newly designated family *Alloherpesviridae* (5,6) (Figure 2, panel A). *Alloherpesviridae* viruses infect fish and amphibians. The common ancestor of this family is thought to have diverged from the common ancestor of the family *Herpesviridae* (herpesviruses that infect reptiles, birds, and mammals) (6). According to phylogenetic analysis of specific genes, the family *Alloherpesviridae* seems to be subdivided into 2 clades (6) (Figure 2, panel B). The first clade comprises anguillid and cyprinid herpesviruses, which possess the largest genomes in the order Herpesvirales (245–295 kb). The second clade comprises ictalurid, salmonid, acipenserid, and ranid herpesviruses, which have smaller DNA genomes (134–235 kb).

### Structure

**Figure 3:**
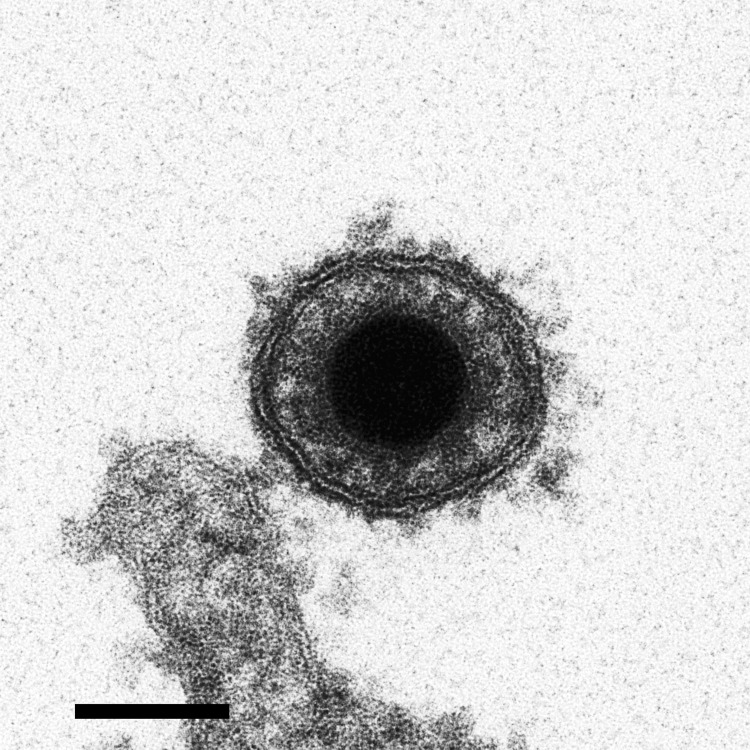
Electron micrograph image of cyprinid herpesvirus 3 virion. Scale bar = 100 nm. Adapted with permission from Mettenleiter et al. (7).

The CyHV-3 structure is typical of viruses of the order Herpesvirales. An icosahedral capsid contains the genome, which consists of a single, linear, double-stranded DNA molecule. The capsid is covered by a proteinaceous matrix called the tegument, which is surrounded by a lipid envelope derived from host cell trans-golgi membrane (7) (Figure 3). The envelope contains viral glycoproteins (3). The diameter of the entire CyHV-3 particle is 170–200 nm (3,8).

### Molecular Structure

#### Genome

The genome of CyHV-3 is a 295-kb, linear, double-stranded DNA molecule consisting of a large central portion flanked by two 22-kb repeat regions, called the left and right repeats (9). The genome size is similar to that of CyHV-1 but larger than that of other members of the order Herpesvirales, which are generally 125–240 kb.

The CyHV-3 genome encodes 156 potential protein-coding open reading frames (ORFs), including 8 ORFs encoded by the repeat regions. These 8 ORFs are consequently present as 2 copies in the genome (9). Five families of related genes have been described: ORF2, tumor necrosis factor receptor, ORF22, ORF25, and RING families. The ORF25 family consists of 6 ORFs (ORF25, ORF26, ORF27, ORF65, ORF148, and ORF149) encoding related, potential membrane glycoproteins. The expression products of 4 of the sequences were detected in mature virions (ORF25, ORF65, ORF148, and ORF149) (10). CyHV-3 encodes several genes that could be involved in immune evasion processes, such as ORF16, which codes for a potential G-protein coupled receptor; ORF134, which codes for an IL-10 homolog; and ORF12, which codes for a tumor necrosis factor receptor homolog.

Within the family *Alloherpesviridae*, anguillid herpesvirus 1 is the closest relative of CyHV-3 that has been sequenced (11). Each of these viruses possesses 40 ORFs exhibiting similarity. Sequencing of CyHV-1 and CyHV-2 will probably identify more CyHV-3 gene homologs. The putative products of most ORFs in the CyHV-3 genome lack obvious relatives in other organisms; 110 ORFs fall into this class. Six ORFs encode proteins with closest relatives in virus families such as *Poxviridae* and *Iridoviridae* (9). For example, CyHV-3 genes such as B22R (ORF139), thymidylate kinase (ORF140), thymidine kinase (ORF55), and subunits of ribonucleotide reductase (ORF23 and ORF141) appear to have evolved from poxvirus genes (9). Neither thymidylate kinase nor B22R has been identified previously in a member of the order Herpesvirales.

Three unrelated strains of CyHV-3, isolated in Israel (CyHV-3 I), Japan (CyHV-3 J), and the United States (CyHV-3 U), have been fully sequenced (9). Despite their distant geographic origins, these strains exhibit high sequence identity. Low diversity of sequences among strains seems to be a characteristic of the CyHV-3 species. Despite this low diversity, molecular markers enabling discrimination among 9 genotypes (7 from Europe and 2 from Asia) have been identified (12).

Because CyHV-3 possesses the largest genome among members of the order Herpesvirales, it provides a model for mutagenesis of large DNA viruses. Recently, the CyHV-3 genome was cloned as a stable and infectious bacterial artificial chromosome, which could be used to produce CyHV-3 recombinants (13).

#### Structural Proteome

The structural proteome of CyHV-3 was recently characterized by using liquid chromatography tandem mass spectrometry (10). A total of 40 structural proteins, comprising 3 capsid, 13 envelope, 2 tegument, and 22 unclassified proteins, were described. The genome of CyHV-3 possesses 30 potential transmembrane-coding ORFs (9). With the exception of ORF81, which encodes a type 3 membrane protein expressed on the CyHV-3 envelope (10,14), no CyHV-3 structural proteins have been studied. ORF81 is thought to be one of the most immunogenic (major) membrane proteins of CyHV-3 (14).

### In Vitro Replication

CyHV-3 is widely cultivated in cell lines derived from koi fin, *C. carpio* carp brain, and *C. carpio* carp gill (3,4,8,15–17) (Table 1). Other cell lines have been tested, but few have been found to be permissive for CyHV-3 infection (Table 1).

**Table 1:** Cyprinid herpesvirus 3–susceptible cell lines

Cell type (cell line)	Cytopathic effect (reference)
*Cyprinus carpio* brain (CCB)	Yes (8,15)
*C. carpio* gill (CCG)	Yes (8)
*Epithelioma papulosum* cyprinid (EPC)	No (3,4,15,16); Yes (8)
Koi fin (KFC, KF-1)	Yes (3,4,15,17)
Carp fin (CFC, CaF-2)	Yes (8)
Fathead minnow (FHM)	No (3,15); Yes (16)
Chinook salmon embryo (CHSE-214)	No (16)
Rainbow trout gonad (RTG-2)	No (16)
Goldfish fin (Au)	Yes (15)
Channel catfish ovary (CCO)	No (15)
Silver carp fin (Tol/FL)	Yes (15)

The CyHV-3 replication cycle was recently studied by use of electron microscopy (7). Its morphologic stages suggested that it replicates in a manner similar to that of members of the family *Herpesviridae.* Capsids leave the nucleus by budding at the inner nuclear membrane, resulting in formation of primary enveloped virions in the perinuclear space. The primary envelope then fuses with the outer leaflet of the nuclear membrane, thereby releasing nucleocapsids into the cytoplasm. Final envelopment occurs by budding into trans-golgi vesicles. Because CyHV-3 glycoproteins have little or no similarity with those of members of the family *Herpesviridae*, identification of the CyHV-3 glycoproteins involved in entry and egress will require further study.

**Figure 4:**
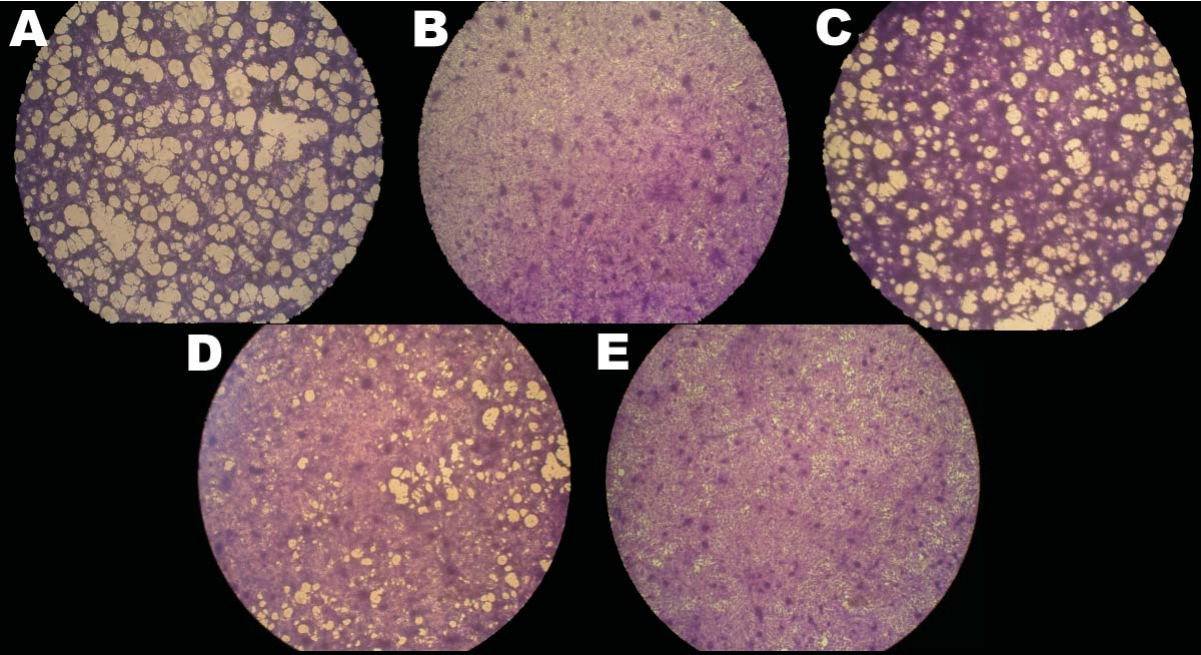
Effects of temperature on cyprinid herpesvirus 3 replication in Cyprinus carpio carp brain cells. After infection, cells were kept at 22°C (A) or shifted to 30°C (B–D); some cells were returned to 22°C at 24 hours (C) or 48 hours (D) postinfection. Uninfected control cells (E) and infected cells at 9 days postinfection were fixed, stained, and photographed. Viral replication was highest in cells maintained at 22°C and lowest in those maintained at 30°C. Original magnification ×20. Adapted with permission from Dishon et al. (18).

Because fish are poikilotherms and because CyHV-3 only affects fish when the water temperature is 18°C–28°C, the effect of temperature on CyHV-3 replication growth in vitro has been investigated. Replication in cell culture is restricted by temperature; optimal viral growth is at 15°C–25°C. Virus propagation and virus gene transcription are turned off when cells are moved to a nonpermissive temperature of 30°C (18). Despite the absence of detectable virus replication, infected cells maintained for 30 days at 30°C preserve infectious virus, as demonstrated by viral replication when the cells are returned to permissive temperatures (18) (Figure 4). These results suggest that CyHV-3 can persist asymptomatically for long periods in the fish body when the temperature prevents virus replication; bursts of new infection occur after exposure to permissive temperatures.

## Disease Caused by CyHV-3

### History

In 1998, the first mass deaths of common and koi carp were reported in Israel and the United States (3). However, analyses of samples from archives determined that the virus had been in wild common carp since 1996 in the United Kingdom (19). Soon after the first report, outbreaks of CyHV-3 were identified in countries in Europe, Asia, and Africa. Currently, CyHV-3 has been identified everywhere in the world except South America, Australia, and northern Africa (20). Worldwide, CyHV-3 has caused severe financial and economic losses in the koi and common carp culture industries.

### Host Range

Common and koi carp are the only species known to be affected by CyHV-3 infection (21). Numerous fish species, cyprinid and noncyprinid, were tested for their ability to carry CyHV-3 asymptomatically and to spread it to unexposed carp (21–23) (Table 2). CyHV-3 DNA was recovered from only 2 other fish species: goldfish and crucian carp. Cohabitation experiments suggest that goldfish, grass carp, and tench can carry CyHV-3 asymptomatically and spread it to unexposed common carp. Hybrids (koi–goldfish and koi–crucian carp) die of CyHV-3 infection (24).

**Table 2:** Fish tested for cyprinid herpesvirus 3 infection*

Species (common name)	Inoculated fish (reference)			Carp deaths during cohabitation (reference)
	DNA	Protein	Clinical signs	
*Carassius auratus* (goldfish)	Yes (23)	Yes (23)	No (21); Yes (23)	No (21); Yes (22)
*Ctenopharyngodon idella* (grass carp)	NT	NT	No (21)	No (21); Yes (22)
*Carassius carassius* (crucian carp)	NT	NT	NT	No (22)
*Hypophthalmichthys molitrix* (silver carp)	NT	NT	No (21)	No (21,22)
*Aristichtys nobilis* (bighead carp)	NT	NT	NT	No (22)
*Bidyanus bidyanus* (silver perch)	NT	NT	No (21)	No (21)
*Oreochromis niloticus* (Nile tilapia)	NT	NT	No (21)	No (21)
*Tinca tinca* (tench)	NT	NT	NT	Yes (22)
*Silurus glanis* (sheatfish)	NT	NT	NT	No (22)
*Vimba vimba* (vimba)	NT	NT	NT	No (22)
*Acipenser ruthenus* (sterlet)	NT	NT	NT	No (22)
*Acipenser gueldenstaedtii* (Russian sturgeon)	NT	NT	NT	No (22)
*Acipenser oxyrinchus* (Atlantic sturgeon)	NT	NT	NT	No (22)

*NT, not tested.

### Susceptibity

CyHV-3 affects carp of all ages, but younger fish (1–3 months, 2.5–6 g) seem to be more susceptible to infection than mature fish (1 year, ≈230 g) (16,21). Recently, the susceptibility of young carp to CyHV-3 infection was analyzed by experimental infection (25). Most infected juveniles (>13 days posthatching) died of the disease, but the larvae (3 days posthatching) were not susceptible.

### Pathogenesis

**Figure 5:**
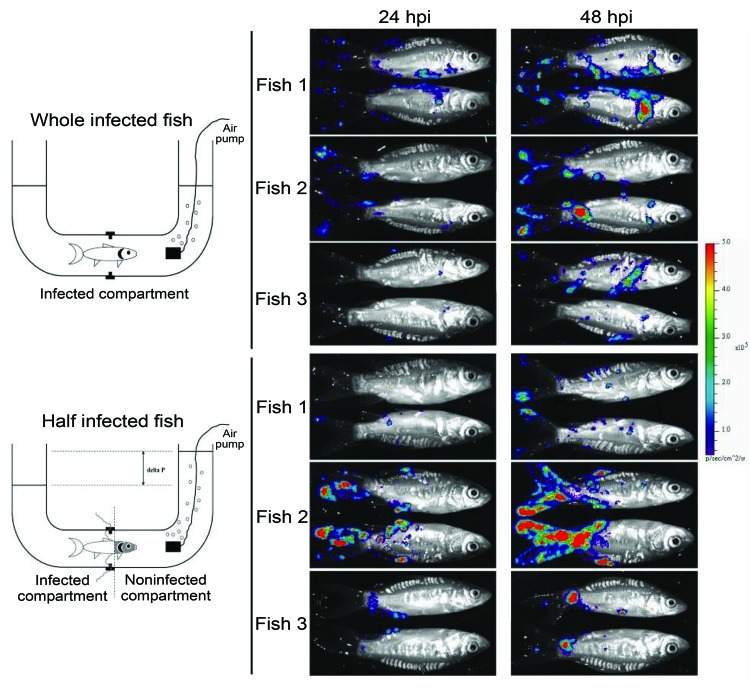
Skin of carp as a portal of entry for cyprinid herpesvirus 3. A schematic representation of the system used to restrict viral inoculation to the fish skin is shown on the left. The lower drawing shows the conditions under which 6 fish were inoculated by restricted contact of the virus with the skin located posterior to the anterior part of the dorsal fin. The upper drawing shows control conditions under which 6 fish were inoculated in the system but without the latex diaphragm dividing the fish body into 2 isolated parts, enabling virus to reach the entire fish body. The fish were infected by bathing them for 24 h in water containing 2 × 10^3^ PFU/mL of a recombinant cyprinid herpesvirus 3 strain able to emit bioluminescence. All fish were analyzed 24 h postinfection (hpi) by bioluminescence imaging. After an additional incubation period of 24 h in individual tanks containing fresh water, they were reanalyzed by bioluminescence imaging at 48 hpi. Three representative fish are shown. The images are shown with standardized minimum and maximum threshold values for photon flux. Adapted with permission from Costes et al. (29).

Several researchers have postulated that the gills might be the portal of entry for CyHV-3 (17, 26–28); however, this hypothesis was recently refuted (29). Bioluminescent imaging and an original system for performing percutaneous infection restricted to the posterior part of the fish showed that the skin covering the fin and body mediated entry of CyHV-3 into carp (29) (Figure 5). This study, together with an earlier study of the portal of entry of a rhabdovirus (infectious hematopoietic necrosis virus) in salmonids (30), suggests that the skin of teleost fish represents an efficient portal of entry for certain viruses. The skin of teleost fish is a stratified squamous epithelium that, unlike its mammalian counterpart, is living and capable of mitotic division at all levels, even the outermost squamous layer. The scales are dermal structures. More extensive studies are needed to demonstrate that the skin is the only portal of entry of CyHV-3 into carp.

After initial replication in the epidermis (29), the virus is postulated to spread rapidly in infected fish, as indicated by detection of CyHV-3 DNA in fish tissues (27). As early as 24 hours postinfection, CyHV-3 DNA was recovered from almost all internal tissues (including liver, kidney, gut, spleen, and brain) (27), where viral replication occurs at later stages of infection and causes lesions. One hypothesis regarding the rapid and systemic dissemination indicated by PCR is that CyHV-3 secondarily infects blood cells. Virus replication in organs such as the gills, skin, and gut at the later stages of infection represents sources of viral excretion into the environment. After natural infection under permissive temperatures (18°C–28°C), the highest mortality rates occur 8–12 days postinfection (dpi) (21). Gilad et al. suggest that death is due to loss of the osmoregulatory functions of the gills, kidneys, and gut (27).

All members of the family *Herpesviridae* exhibit 2 distinct life-cycle phases: lytic replication and latency. Latency is characterized by maintenance of the viral genome as a nonintegrated episome and expression of a limited number of viral genes and microRNAs. At the time of reactivation, latency is replaced by lytic replication. Latency has not been demonstrated conclusively in members of the family *Alloherpesviridae*. However, some evidence supports existence of a latent phase. CyHV-3 DNA has been detected by real-time PCR at 65 dpi in clinically healthy fish (27). Furthermore, the virus persisted in a wild population of common carp for at least 2 years after the initial outbreak (31). Finally, St-Hilaire et al. demonstrated the possibility of a temperature-dependent reactivation of CyHV-3 lytic infection several months after initial exposure to the virus (32). This finding suggests that the temperature of the water could control the outcome of the infection (replicative/nonreplicative). Whether the observations described above reflect latent infection, as described for the family *Herpesviridae*, or some type of chronic infection, remains to be determined. Similarly, the carp organs that support this latent or chronic infection still need to be identified.

### Transmission

Horizontal transmission of CyHV-3 in feces (26) and secretion of viral particles into water (21) have been demonstrated. The skin of carp acts as the portal of entry of CyHV-3 and the site of early replication (29). The early replication of the virus at the portal of entry could contribute not only to the spread of the virus within infected fish but also to the spread of the virus throughout the fish population. As early as 2–3 dpi, infected fish rubbed against other fish or against objects. This behavior could contribute to a skin-to-skin mode of transmission. Later during infection, this mode of transmission could also occur when uninfected fish pick at the macroscopic herpetic skin lesions on infected fish. To date, no evidence of vertical transmission of CyHV-3 has been found.

### Clinical Signs

**Figure 6:**
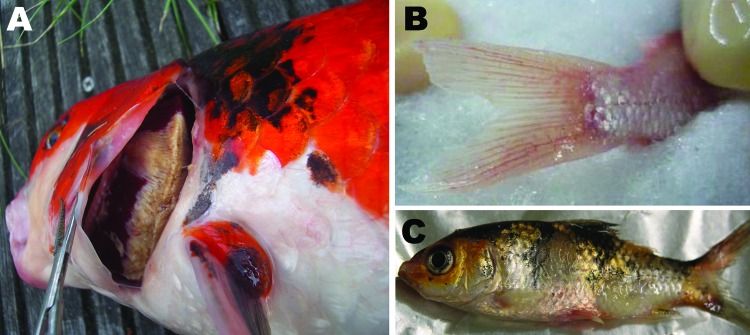
Clinical signs in cyprinid herpesvirus 3–infected fish. A) Severe gill necrosis; B) hyperemia at the base of the caudal fin; C) herpetic skin lesions on the body and fin erosion.

The first signs appear at 2–3 dpi. The fish exhibit appetite loss and lethargy and lie at the bottom of the tank with the dorsal fin folded. Depending on the stage of the infection, the skin exhibits different clinical signs, such as hyperemia, particularly at the base of the fins and on the abdomen; mucus hypersecretion; and herpetic lesions (Figure 6). The gills frequently become necrotic and hypersecrete mucus, which suffocates the fish. Bilateral enophthalmia is observed in the later stages of infection. Some fish show neurologic signs in the final stage of the disease, when they become disoriented and lose equilibrium (3,19,21).

### Histopathologic Findings

In CyHV-3 infected fish, prominent pathologic changes occur in the gill, skin, kidney, liver, spleen, gastrointestinal system, and brain (3,17,21,28). Histopathologic changes appear in the gills as early as 2 dpi and involve the epithelial cells of the gill filaments. These cells exhibit hyperplasia, hypertrophy, and/or nuclear degeneration (3,17,21,28). Severe inflammation leads to the fusion of respiratory epithelial cells with cells of the neighboring lamellae, resulting in lamellar fusion (17,28). In the kidney, a weak peritubular inflammatory infiltrate is evident as early as 2 dpi and, along with blood vessel congestion and degeneration of the tubular epithelium in many nephrons, increases with time (17). In the spleen and liver, splenocytes and hepatocytes, respectively, are the most obviously infected cells (28). In brain of fish that showed neurologic signs, congestion of capillaries and small veins are apparent in the valvula cerebelli and medulla oblongata, associated with edematous dissociation of nerve fibers (28).

### Diagnosis

Diagnosis of CyHV-3 infection is described elsewhere (20). Suspicion of CyHV-3 infection is based on clinical signs and histopathologic findings. Since initial isolation of CyHV-3 in 1999, complementary diagnostic methods have been developed. Virus isolation from infected fish tissues in cell culture (*C. carpio* carp brain and koi fin cells) was the first method to be developed (3). This time-consuming approach is still the most effective method for detecting infectious particles during an outbreak of CyHV-3 infection. A complete set of techniques for detecting viral genes—including PCR (20), nested PCR (33), TaqMan PCR (27), and loop-mediated isothermal amplification (34)—has been developed. Real-time TaqMan PCR has been used to detect CyHV-3 in freshwater environments after concentration of viral particles (2). Finally, ELISAs have been developed to detect specific anti-CyHV-3 antibodies in the blood of carp (35) and to detect CyHV-3 antigens in samples (17,26).

## Immune Response

Immunity in ectothermic vertebrates differs in several ways from that of their mammalian counterparts. Environmental temperature has drastic effects on the fish immune system. In carp, for example, at <14°C, adaptive immunity is inhibited, but the innate immune response remains functional (36). As mentioned above, host temperature also has an effect on CyHV-3 replication, which can occur only at 18°C–28°C. In carp that are infected and maintained at 24°C, antibody titers begin to rise at ≈10 dpi and plateau at 20–40 dpi (37). In the absence of antigenic reexposure, the specific antibodies gradually decrease over 6 months to a level slightly above or comparable to that of unexposed fish. Although protection against CyHV-3 is proportional to the titer of specific antibodies during primary infection, immunized fish, even those in which antibodies are no longer detectable, are resistant to a lethal challenge, possibly because of the subsequent rapid response of B and T memory cells to antigen restimulation (37).

## Prophylaxis and Control

For CyHV-3 control, 3 approaches are being developed. They are 1) management and commercial measures to enhance the international market of certified CyHV-3–free carp and to favor eradication of CyHV-3, 2) selection of CyHV-3–resistant carp, and 3) development of safe and efficacious vaccines.

### Selection of CyHV-3–Resistant Carp

Carp resistance to CyHV-3 might be affected by host genetic factors. Shapira et al. demonstrated differential resistance to CyHV-3 (survival rates 8%–60%) by cross-breeding sensitive domesticate strains and a resistant wild strain of carp (38). Further supporting the role of host genetic factors in CyHV-3 resistance, major histocompatibility class II genes were recently shown to affect carp resistance (39).

### Vaccination of Carp

Soon after the characterization of CyHV-3, a protocol to induce a protective adaptive immune response in carp was developed. This approach is based on the fact that CyHV-3 induces fatal infections only when the water temperature is 18°C–28°C.

According to this protocol, healthy, uninfected fish are exposed to CyHV-3 infected fish for 3–5 days at permissive temperature (22°C–23°C) and then transferred for 30 days to ponds at a nonpermissive temperature (≈30°C). After this procedure, 60% of fish become resistant to further challenge with CyHV-3 (4). Despite its ingenuity, this method has several disadvantages: 1) increasing the water temperature to 30°C makes the fish more susceptible to secondary infection by other pathogens and requires a large amount of energy in places where the water is naturally cool; 2) the protection is observed in only 60% of fish; 3) carp that are “vaccinated” by using this protocol have been exposed to wild-type virulent CyHV-3 and could therefore represent a potential source of CyHV-3 outbreaks if they later come into contact with an unexposed carp.

Attenuated live vaccine appears to be the most appropriate for mass vaccination of carp. Attenuated vaccine candidates have been produced by successive passages in cell culture (4). The vaccine strain candidate was further attenuated by UV irradiation to increase the mutation rate of the viral genome (4,37). A vaccine strain obtained by this process has been produced by KoVax Ltd. (Jerusalem, Israel) and has been shown to confer protection against a virulent challenge. However, this vaccine is available in only Israel and has 2 main disadvantages: 1) the molecular basis for the reduced virulence is unknown, and consequently, reversions to a pathogenic phenotype cannot be excluded; and 2) under certain conditions, the produced attenuated strain could retain residual virulence that could be lethal for a portion of the vaccinated fish (3*7*).

An inactivated vaccine candidate was described by Yasumoto et al. (40). It consists of formalin-inactivated CyHV-3 trapped within a liposomal compartment. This vaccine can be used for oral immunization in fish food. Protection efficacy for carp is 70% (40).

## Conclusions

Because CyHV-3 causes severe financial losses in the common carp and koi culture industries worldwide, it is a useful subject for applied science. Safe and efficacious vaccines adapted to mass vaccination of carp and efficient diagnostic methods need to be developed. Several aspects of CyHV-3 make it also useful for fundamental science. These aspects are its large genome, the relationship between CyHV-3 infectivity and temperature, and the low similarity between CyHV-3 genes and the genes of other members of the order Herpesvirales that have been studied. Further studies are needed to identify the roles of CyHV-3 genes in viral entry, egress, and disease pathogenesis.
